# Making end-of-life health disparities in the U.S. visible through family bereavement narratives

**DOI:** 10.1016/j.pecinn.2024.100276

**Published:** 2024-03-25

**Authors:** Cassidy Taladay-Carter

**Affiliations:** Department of Communication Studies, University of Nebraska—Lincoln, 356 Louise Pound Hall, Lincoln, NE 68588, USA

**Keywords:** End-of-life, Narratives, Health disparities, Family, Terminal illness, Palliative care and hospice

## Abstract

**Objective:**

End-of-life experiences can have important implications for the meaning-making and communication of bereaved family members, particularly due to (in)access to formal healthcare services (i.e., palliative care and hospice). Grounded in Communicated Narrative Sense-Making theory, this study extends knowledge about how the stories told about end-of-life by bereaved family members affect and reflect their sense-making, well-being and importantly, potential disparities in end-of-life care.

**Methods:**

Semi-structured interviews with 25 bereaved individuals were conducted regarding their experiences with the terminal illness and death of an immediate family member. Using a framework of family bereavement narratives, a cross-case data analysis demonstrated qualitative patterns between (in)access to end-of-life care and how participants framed bereavement stories.

**Results:**

Four themes illustrated the continuum of communication that families engaged in when making sense of end-of-life experiences, including *reflections on silence*, *tempered frustrations, comfort with care*, and *support from beyond*.

**Innovation:**

This innovative qualitative connection between family members' bereavement stories and end-of-life care emphasizes the importance of employing a health equity lens within hospice and palliative care, especially in addressing the important aim of comprehensively supporting families even when illness ends. This study demonstrates that access to, quality of, and imagining beyond current structures for EOL may be vital factors for facilitating effective sense-making for the dying and their family systems.

**Conclusion:**

These findings illustrate the potential interconnections between (in)access to end-of-life care, sense-making, and communication for individuals and families experiencing terminal illness and bereavement.

## 1 Introduction

As institutionalized end-of-life (EOL) care (i.e., palliative care, hospice) in the U.S. rises, such services are increasingly tasked with supporting dying individuals and their families [1]. These programs frequently facilitate this support for patients, family members, and healthcare providers (HCPs) through symptom management and collaborative communication [2]. Notably, EOL services also promote well-being by facilitating spaces for *sense-making*, whereby individuals engage in efforts to find, create, or reconstruct meaning of EOL [3,4]. One important way that individuals make meaning of difficult illness experiences is through telling stories, theoretically termed *communicated narrative sense-making* (CNSM, [5]). EOL programs advance CNSM around illness and death due in part to the communicative work of social workers, therapists, grief groups, and advance care planning processes [2,6]. The presence of these services within EOL care structures may foster sense-making processes that enhance adjustment and well-being for patients and families alike [5].

It follows that the stories that bereaved families tell about EOL, death, and grief, may also reveal much about their health and well-being. Taladay-Carter and Koenig Kellas [7] investigated bereavement stories told by immediate family members upon a relative's death due to terminal illness, identifying three time-oriented narratives characterizing family bereavement. First *past stories* focused on recollection, appreciation of, and respect for the memory of deceased family members; next, *present stories* illustrated ongoing tensions of family grief, such as the challenges of re-defining family upon death and simultaneous connection and meaning found in bereavement; and finally, *future stories* emphasized lessons learned upon bereavement, including how caretaking and death shaped storytellers' beliefs and relationships with others. Notably, the focus on time in family bereavement narratives aligns with the core purpose of writing one's life story—to incorporate the reconstructed past, perceived present, and anticipated future, and thus provide a sense of unity and purpose for life's challenges [8,9]. While extant research emphasizes that storytelling around these challenges can be a therapeutic or supportive sense-making process, not all stories are told the same, nor does it indisputably benefit health and well-being [5]. In other words, there may be important factors that shape how the content of these past, present, and future bereavement stories are told and *framed*, and thus how they impact grieving families.

The purpose of this study is to explore how one factor—access to EOL care—might shape the content, framing, and associated health and well-being stemming from these stories. Though hospice and palliative care may often facilitate CNSM, the extent to which dying patients and their families can(not) access EOL services that would do so matters. Scholars have previously explored how palliative care can impact family storytelling [10] without an explicit focus on the impact of *(in)*access on sense-making. Yet when families are left to make sense of their experiences with death and loss, their access to EOL care is an important factor to consider. Relatedly, inequities in the larger healthcare system limit the benefits of EOL services for many [11]. Numerous individual (i.e., stigma, limited information) and structural (i.e., geography, cost, quality of services) barriers affect who has access to care and to what extent it is meaningful in their lives [12,13]. EOL care services are disproportionately developed and used by white, Northern European descent groups, rarely attending to identities and contexts that are particularly important in the face of death (e.g., religion, race/ethnicity, socioeconomic status) [14]. These societal and structural factors have contributed to disparities in desired care and satisfaction with care received between those with EOL services and those without [15]. Often, this includes families of color, of diverse sexual orientation and (dis)ability, and those in rural settings [14]. Researching benefits and challenges related to these services, especially for the valuable task of EOL and bereavement sense-making, provides an impetus for improving the access and quality of current EOL care structures while also recognizing their inherent limitations.

CNSM theory [5] is a useful framework for investigating how the content and framing of family bereavement stories may reflect and affect health and well-being when families do (not) have access to supportive EOL care. CNSM's first proposition asserts that the content of individual stories reveals individual, relational, and intergenerational *meaning-making, values, and beliefs*, defined as the significance, importance, worth, and/or principles communicated through the content of the stories people tell Koenig Kellas & Taladay-Carter, [16]. Further, CNSM's second proposition posits that story content that is more positively framed will be more positively related to well-being. CNSM's propositions may inform the impact of significant and lasting bereavement stories, and how various framing (i.e., more negative, more positive) of story content may result in varying outcomes for storytellers [5].

This investigation is thus grounded in propositions 1 and 2 of CNSM theory [5], extending [7] findings by analyzing how family bereavement narratives align with patient and family (in)access to EOL care. Thus, the guiding research question is:

RQ: What patterns can be identified between (a) types of family bereavement narratives and (b) (in)access to EOL care services?

## 2 Methods

### 2.1 Author reflexivity

The author is a white, American cisgender female graduate student with personal experiences around terminal illness, bereavement, and grief. These intersecting identities provide multiple privileges in relation to these data, as well as lenses that guided the analysis, writing, and contributions of this study, in the context of storytelling about EOL care and inequities.

### 2.2 Participants

Participants (*N* = 25) were at least 19 years of age, resided within the U.S., and had experienced the terminal illness and corresponding death of an immediate family member (i.e., spouse, sibling, parent, child) at least one year before the study. Participants were primarily white women (*n* = 17) averaging 43.8 years of age (*SD* = 12.9 years) and geographically dispersed across the U.S. In relation to the deceased, participants were spouses (*n* = 7), children (*n* = 16), and siblings (*n* = 2). Most held a degree beyond high school (*n =* 22, 88%) and indicated household incomes ranging from $20,000 to $29,000 (*n* = 2) to $100,000 or more (*n* = 15). Terminal diagnoses of family members included cancers (*n =* 18), pulmonary fibrosis (*n* = 3), heart diseases (*n =* 3), and immune disorders (*n* = 1).

### 2.3 Procedures

Following IRB approval, participants were recruited online (e.g., Reddit, Facebook) via purposive and snowball sampling [17]. Interested individuals provided informed consent and demographics via a Qualtrics survey. Interviews were scheduled and then conducted on Zoom due to COVID-19 between January and February 2021. A semi-structured narrative interview protocol guided participants to tell their family stories of terminal illness and death [18]. Participants were instructed to “Tell the story of your family's terminal illness and bereavement” and prompted with follow-up questions such as “How did EOL services impact your family's experience of illness and death?” and “What were your family's interactions with services/HCPs like?” Interviews averaged 78.5 min (*SD* = 25.2 min) and were video-recorded and auto-transcribed, resulting in 774 double-spaced pages of data. The author manually cleaned transcripts for errors and disfluencies and replaced all identifying information with pseudonyms.

### 2.4 Data analysis

These data come from a larger study on narratives in family bereavement (see Taladay-Carter & Koenig Kellas) [7]. Building from Taladay-Carter and Koenig Kellas' [7] typology of family bereavement narratives and in line with CNSM theory [5], the present analysis aimed to evaluate the same story content to determine whether types of bereavement narratives reflected the (in)access of dying individuals and family members (the participants). Participants' stories were preserved as intact units and assigned one of three narrative types (i.e. past, present, future) stemming from an initial narrative thematic analysis ([18]; see Taladay-Carter & Koenig Kellas) [7]. Then, participants' Qualtrics responses to the question “Did you have access to EOL care services?” were used to categorize families that *did* (answered yes) or *did not* (answered no) have access to palliative care or hospice; interviews further inquired about the extent and quality of care.

Story type and EOL (in)access were then synthesized into a *cross-case data matrix*, a qualitative analysis method involving the thematic comparison of similarities and differences in data to highlight potentially meaningful overlap or patterns [19]. After each participant's interview transcript was coded for (a) family bereavement story and (b) (in)access to EOL care, the individual cases were organized into a 3 × 3 cross-case data matrix to display qualitative patterns of participants' experiences at EOL. The transcripts with overlap (e.g., those with *past* stories/access to EOL care, *present* stories/no EOL care) were then compared to generate themes characterizing EOL care and communication [20] (see Table 2). Findings were verified through a data conference [21], seven member checks [22], and exemplar identification [23]. (See Table 1.)

**Table 1:** Participant demographics.

Characteristic	N (%)
Age	
25–34	9 (36)
35–44	4 (16)
45–54	9 (36)
55–64	1 (4)
65–74	2 (8)

Race/Ethnicity	
White	21 (68)
African American or Black	2 (8)
Biracial or Multiracial	2 (8)

Gender Identity	
Female	17 (68)
Male	8 (32)

Household Annual Income	
$20,000–$29,000	2 (8)
$30,000–$39,000	1 (4)
$40,000–$49,000	3 (12)
$50,000–$59,000	0 (0)
$60,000–$69,000	2 (8)
$70,000–$79,000	1 (4)
$80,000–$89,000	1 (4)
$90,000–$99,000	1 (4)
$100,000 or more	15 (60)

Access to EOL Services	
Indicated Access (Yes)	19 (76)
Indicated No Access (No)	6 (24)

Educational Attainment	
High School or equivalent	3 (12)
Associate degree	2 (8)
Bachelor's degree	7 (28)
Graduate degree	13 (52)

Religion	
Protestant Christian	10 (40)
Agnostic (spiritual/believe in something)	5 (20)
Atheist/Nothing	5 (20)
Church of Jesus Christ of Latter-Day Saints	2 (8)
Roman Catholic	2 (8)
Jewish	1 (4)

Family Member Diagnosis	
Cancer	18 (72)
Pulmonary Fibrosis/COPD	3 (12)
Heart Disease	3 (12)
Immune Disorder	1 (4)

**Table 2:** Summary of Cross-Case Matrix Themes.

Theme Name	Theme Description	Bereavement Story Type	Access to EOL Care	% of Participants
Reflections on Silence	Faced resistance from families and HCPs in EOL communication; Navigated absence of formal outlets by sharing stories of deceased	Past	No	12%
Tempered Frustrations	Held unanswered questions and anguish about EOL due HCPs; Reframed small joys and connection in death and dying	Present	No	12%
Comfort with Care	Felt confident and empowered to discuss the deceased and shared family moments; Relied on communication resources to overcome HCP-related challenges	Past + Present	Yes	60%
Support from Beyond	Experienced profound spiritual or relational moments, spurring support and/or generativity toward others	Future	Yes	16%

## 3 Results

In addressing the research question regarding patterns between types of family bereavement narratives and (in)access to EOL services, the cross-case data analysis resulted in four themes illustrating a continuum of care and communication strategies that constituted how family members made sense of terminal illness and bereavement. The first two themes, *reflections on silence* and *tempered frustrations*, articulated the unique aspects of the stories constructed by participants who indicated that they did not have access to EOL services. The following two themes, *comfort with care* and *support from beyond*, described how participants narrated experiences when EOL care was available.

### 3.1 Reflections on silence

The first theme, *reflections on silence*, illustrated the immense resistance that participants faced from family, community members, and HCPs in talking about death, dying, and grieving. This theme represented those three participants without access to EOL care and who told *past* stories. In *past* stories, participants primarily made sense of illness and bereavement by honoring and reflecting on the identity or legacy of the deceased. While past stories signal value in continuing bonds with the deceased, the content of these past stories reflected that participants encountered silence when they sought to stay connected to and share the stories of their deceased loved ones. Upon her mother's death, Leslie reflected:

It's just one of those things. Not having [my mom] around… nobody talked about her for many, many years. It was almost like, taboo. You don't talk about grandma because I think it was too emotional for people. None of my family knew how to mourn her. Nobody did. It took a really, really long time.

In addition to family-level silence, participants also acknowledged the absence of formal outlets for engaging in open communication about the identities, characteristics, or stories of the deceased. For instance, Diana noted, “I'm realizing that my family just didn't have anyone to help us through coping with [mom's] death.” However, participants enacted agency in response to said silence, taking on the responsibility for initiating family discussions about the deceased and using the story of their deceased to break the compounding silences they experienced. Without EOL resources (e.g., support groups, mental health professionals), participants framed these stories around the ways they carefully navigated the silences they encountered at individual, family, and structural levels. All three reflected on how they have shouldered the immense responsibilities involved in remembering, honoring, and dignifying their loved ones upon bereavement, regardless of the obstacles they face.

### 3.2 Tempered frustrations

The second theme, *tempered frustrations*, highlighted participants' reactions to the fluctuating, confusing nature of communication around death and dying. Participants specifically pointed to stressors stemming from interactions with HCPs who dismissed or could not provide needed EOL support. This theme represented those three participants without access to EOL care and who told *present* stories to make sense of their experiences. *Present* stories are characterized by the tensions spurred by illness and death (e.g., conflicts between family members about the dying process versus appreciating moments spent together as a family; feelings of despair versus recognizing beauty in life and death). Accordingly, participants experienced anguish in unanswered questions and frustrations with HCPs and healthcare systems while also emphasizing the small joys and beautiful moments—those moments often tempering their reactions to EOL challenges. For instance, Oliver described competing emotions when his dying wife's doctors made undisclosed care decisions that did not align with his own preferences. While his story was told retrospectively, the major takeaways illustrated lingering feelings of disappointment and distrust in care providers *and* joy in moments shared with his partner:

When [my wife] was in remission, that was a really joyful moment in time. We got engaged during that brief interlude between treatment plans. That was another joyful moment and then when the cancer came back, it wasn't a huge surprise right, because Stage 4… you never really care if it's gotten to the point where Stage 4 means. You know, kind of floating all around. And the best you can hope for is it, like, holding it at bay for long enough to live in naturally long life. So again, we weren't shocked when it came back just disappointed, I suppose, but the doctors, of course, had their plans that we didn't quite understand and I still don't.

Oliver's story demonstrated how his frustration at a lack of supportive EOL care was mellowed by his recognition of moments that mattered at the end of his wife's life. Ultimately, per their *present* stories, all three participants highlighted how these experiences still impacted them daily, such as Oliver's lack of trust in HCPs. Another participant, Monique, continued to feel a sense of responsibility for keeping her family united upon her sister's death due to limited medical support and estrangement from important family members. In her story, Monique reframed these challenges:

I think when you lose a sibling, you know, you hold closer, it brings you much closer to your other siblings. You know, I think it did have an impact. Like you just never know. Life's too short. You just never know when it is our time.

Monique's story also emphasized that when the necessary EOL information or support was not provided by the medical system, *present* stories notably (re)framed toward the more positive aspects of what participants and families had control over (e.g., appreciation of family strengths, time together).

In total, six participants did not access EOL services; three told *past* stories and three told *present* stories. When their access to EOL care was examined alongside the stories they told (i.e., cross-case analysis), multiple levels of barriers to supportive communication, quantity, and quality of care emerged in themes of *reflections on silence* and *tempered frustrations*.

Next, two themes generated when participants had access to EOL services and told their stories (*comfort with care* and *support from beyond*) are discussed. Notably, the number of participants in this study who indicated that their family member accessed EOL care was much greater (*n* = 19) than those who did not (*n* = 6). However, evaluating patterns between story content of those with EOL care reflects the roles that EOL services played in participants' sense-making and coping.

### 3.3 Comfort with care

The third theme, *comfort with care*, illustrated how participants relied on family communication and other resources (i.e., social capital) to feel supported in EOL care, even when facing challenges with HCPs. This theme captured the experiences of fifteen participants whose families had access to palliative care and/or hospice; seven of these participants told *past* stories and eight told *present* stories. The cross-case data analysis revealed important similarities between these participants' EOL experiences that could be enveloped under the same theme, *comfort with care*. All fifteen participants had access to formal support resources at EOL, yet still faced complications. For example, Patty expressed her disappointment in her mother's hospice doctor. She told a *past* story, highlighting her mother's worth and dignity:

I felt like if she had been supported differently, I would have maybe felt like she could have been more comfortable for longer and then still known she could choose the time [to die]. So she really struggled the last few weeks and really went into herself, like didn't talk to me very much.

Patty continued, indicating that she engaged knowledge from past caretaking experiences to inform her mother's EOL and family's communication upon bereavement:

I think we felt lucky that it—it wasn't easy—but it was different for us than some other families who don't talk about death and who, so much in our culture makes birth and death something that happens out of our sight. It happens in the hospital or it happens in a nursing home. It's away because it's dirty or unclean or uncomfortable… For our family, those things have always been part of it.

Though there were similarities between those telling *past* and *present* stories in this theme, there were also unique defining characteristics between how *comfort with care* manifested. Specifically, those like Patty who told *past* stories expressed comfort communicating about death and dying as it pertained to the deceased individual's life and identity—relying on relationships with the deceased to feel empowered in caregiving and opening spaces for family communication about loss. Alternatively, participants telling *present* stories noted a lack of time with EOL care (i.e., less than one week on average) and a limited ability to engage with available EOL resources. Such incomplete EOL care also impacted bereavement, as when Lisa said:

I felt let down. I never knew what was going on. I was never made aware that there [was] care available afterwards. I think I ended up getting the most emotional support in the whole situation from the undertaker at the crematorium.

Despite limitations, these participants emphasized how quality time spent in hospitals or at home with their families was central to their *present* stories. Overall, both sets of participants (i.e., *past* and *present* storytellers) with access to EOL care expressed notable comfort in communicating about illness, death, and bereavement. These storytellers emphasized a *comfort with care* (and communication) that other groups of participants did not.

### 3.4 Support from beyond

The final theme, *support from beyond*, featured monumental spiritual or relational events during illness and/or dying which induced profound, ongoing support for participants within and beyond their families. This theme represented the four participants with access to EOL care and who told *future* stories. *Future* stories are characterized by changing belief systems (i.e., religion/spirituality, transcendence) and generative contributions to the future well-being of others. Accordingly, all four participants storied an experience that they deemed particularly profound or spiritual, and which played an important part in EOL care.

For example, when his husband lay in a coma with mounting pressure from HPCs to remove life-prolonging ventilation, Jackson stumbled upon an old friend—whom he had lived with on another continent nearly 20 years prior—just steps outside of the hospital room. Despite identifying as an atheist, Jackson discussed the “kismet… a reckoning of scales” he felt when his long-lost friend told him:

“I'm going to come and find you a little bit. I am a respiratory therapist here and… I'm going to talk you through the whole process”… which was something that not even palliative care necessarily did that they should have done, even though they were trying to be very supportive. But having someone who I have a history with, who I love, who I was very good friends with in [country], was super lovely.

Jackson was profoundly impacted by how his old friend “opened his heart” in these ways. He connected this moment with the immense support of the palliative care team, friends, and family, all stemming from the peace and confidence gained from the “kismet” encounter. Another participant, Janae, attributed the excellent EOL care that her father received as a major motivator for connecting with other grieving people. Her profound experience came in the form of leading a student grief group at the middle school she worked at soon after her father's death. Janae said:

I don't know that if I hadn't, if my dad hadn't just died at that point, if these young women hadn't been available to put me in their grief group, I don't believe that my life would have been anywhere the same. So to me that's life changing, and [my father's death] was, it was a loss, it was, it was. But there were… some compensations for it… There was a way of not making it all right, right, but making use of what happened to go on.

Participants like Janae and Jackson, who had access to supportive EOL care and framed their stories in the *future*, described these striking moments and how they related to high-quality EOL care. They made sense by looking toward the future, especially how they might reciprocate the care they received for others.

## 4 Discussion and conclusion

### 4.1 Discussion

In summary, four themes were generated through cross-case data analysis [19] to illustrate patterns between participants' (in)access to EOL services and family stories they told upon bereavement. These themes included (a) *reflections on silence*, (b) *tempered frustrations*, (c) *comfort with care*, and (d) *support from beyond* and illustrated a vast continuum of communication experiences around death and dying. Importantly, the cross-case matrix demonstrated how families with access to EOL services experienced *qualitative differences* in making sense of terminal illness and death, such as in feeling more open and empowered to communicate about and within death and dying (*comfort with care*) and desiring to give back based on excellent care they received (*support from beyond*). Participants without access to EOL services experienced mounting silence and tension with family and HCPs that they navigated on their own in a variety of ways (*reflections on silence*, *tempered frustrations*). Accordingly, these themes highlighted important ways that EOL may impact interactions between individuals and healthcare structures. Further, these findings support CNSM theory's propositions related to the significance of the stories that we tell and the ways that those stories are (re)framed relate to health and well-being [5]. This study also contributes to conceptualizing CNSM's concept of health and well-being beyond quantitative measures, with themes illustrating qualitative outcomes or consequences of CNSM processes.

First, terminal illness and bereavement are difficult life experiences that demand sense-making Trees & Koenig Kellas [24]. Stories about difficulties provide a window into the experiences themselves and the meaning making processes involved in them [25]. Decades of research indicate how meaning making—facilitated through storytelling—is central to coping with grief [4]. The present study illustrated this, as participants told stories with similar content (i.e., shared types of stories) though access to supportive care services at EOL reflected different overall takeaways or themes. This may be because participants with hospice and/or palliative care services likely had greater access to “whole person” psychosocial resources such as social workers, therapists, support groups, or spiritual care that supported them in telling and (re)framing the content of their stories in more positive or useful ways [26,5,27]. It may also be that the resources provided through EOL services helped participants make greater meaning of their experiences. For instance, while many participants with and without access to EOL care framed their stories around the *past* and the *present*, only those who accessed palliative care and/or hospice told *future* stories. This may be because those with EOL resources felt less stuck in the tensions (of the present) or looking back at the deceased (of the past), and instead had sense-making resources to support looking toward the future for what they could do with joys, sorrows, and lessons learned. Narrating loss can result in many forms, including rumination, personal growth, and communion, which all signal a different degree to which storytellers meaningfully integrate experiences into their life stories [9]. The current study indicated how participants with EOL care opportunities may have looked to ways they might personally or relationally grow from, rather than ruminate on, challenges.

Furthermore, those who told *past* or *present* stories and had access to EOL care exemplified Wittenberg et al. [10] *comforted journeys* wherein EOL care is embraced early in the illness experience, open awareness of dying is emphasized, and communication (e.g., decision-making, collaboration) takes place across patients, families, and HCPs. Those at other intersections of story types and EOL care did not fit as neatly into Wittenberg et al. [10] other illness journeys (*isolated* or *rescued*), which to some may imply that families are lost or helpless without formal EOL services. However, these findings instead demonstrated how participants creatively navigated circumstances when EOL care was not accessible or low in quality, emphasizing the important role of agency in navigating problematic social structures including emerging health disparities between those with(out) access to EOL care [28]. These findings could be translated for practitioners into more attuned storylistening, looking for identifiable types of stories and supporting patients and/or families in identifying, (re)framing, and connecting over the stories they tell about these difficult experiences [29].

Despite the recognized power of narratives in illness [30] and grief [4] and that EOL services are in key positions to facilitate CNSM, these findings emphasized that without supportive EOL structures, bereaved individuals and families may struggle while *simultaneously* enacting personal agency and strengths. Presently, this manifested as the individual (re)framing of stories by participants around the beauty, connection, and opportunities that their families experienced at EOL and into bereavement. Although CNSM theory's second proposition posits a potentially beneficial relationship between such reframing and well-being [5], individual sense-making and storytelling do not necessarily affect larger systems and structures of healthcare that they are situated within. In the U.S., communities of color (e.g., Black/African American, Indigenous, Latinx) tend to be less satisfied with communication and receive less sense-making support due to disparities in the structures of palliative care and hospice [13]. The connections between stories told and EOL care experience emphasized the importance of health equity lenses in EOL care, particularly the need to increase access to sources of sense-making along with material EOL supports (e.g., medications, respite care) for all.

A primary limitation of this study is the primarily white, wealthy sample whose stories it centers on. In the U.S., whiteness and wealth are tied to healthcare structures and inequities [31]. The intersections of whiteness, wealth, and access to EOL care limit these findings while highlighting the limitations of the current U.S. biomedical system for marginalized communities. Specifically, palliative care and hospice promote culture-specific ideals (e.g., patient-centeredness, involvement of family, advance care planning, [14]) and thus do not serve the needs of all individuals or families. Inequities do not just emerge but are socially constructed, in ways not limited to hospice HCPs disrespecting culturally-based desires related to EOL (e.g., not wanting DNR orders or “aggressive” forms of life-preserving treatment, [32]), language and/or cultural discordance between patients, families, and HCPs [33], and medical racism [34]. Even if individuals and families do have access to these EOL services (as many in this study did), these ideals and practices do not necessarily align with many cultural perspectives on dying, death, and grief. There are many ways to live, to die, and to move forward in grief which do not necessarily involve Western conceptions of medicine or support structures [35]. Scholars and practitioners should continue to engage in community-based, culturally-centered research and practice that prioritizes the voices and lived experiences of communities over the promises of structures not built to support them [36]. Although the EOL structure that dominates in the U.S. is not serving all who experience illness and death, these individuals and families still need spaces for sense-making. In the face of structural barriers, communities often pursue alternative supportive spaces for stories and storytelling, such as churches, community centers, and online spaces [37,38]. Communication and health equity scholarship should uplift sense-making spaces for individuals and families at various levels, within and beyond the blockades of Western biomedical models.

### 4.2 Innovation

This study makes an important contribution to understanding communication at EOL by illustrating qualitative connections between CNSM (i.e., stories and storytelling) and (in)access, as well as potential health disparities, within palliative care and hospice. The cross-case data analysis method is an innovative technique illustrating individual and family experiences with EOL care which is potentially useful for further exploring the communicative roles of health disparities and equity for those on the margins of current structures for supporting meaning-making of illness and death. Furthermore, testing CNSM through these innovative methods is vital for its ongoing development.

## 5 Conclusion

The many benefits and challenges of U.S. EOL services have long been recognized. The stories told by those most affected—bereaved family members—can inform us as to how (in)access impacts the well-being of both those diagnosed with serious illness and those coping with their imminent death. This study supports the important role of CNSM in the continuum of care and communication that families experience when EOL care is present and effective, limited, or altogether absent. Findings suggest we must continue to develop meaningful spaces for stories and storytelling within and beyond current healthcare structures, and how systems can transform to support individuals, families, and communities.

## CRediT authorship contribution statement

**Cassidy Taladay-Carter:** Writing – review & editing, Writing – original draft, Visualization, Validation, Supervision, Software, Resources, Project administration, Methodology, Investigation, Formal analysis, Data curation, Conceptualization.

## Declaration of competing interest

The authors declare that they have no known competing financial interests or personal relationships that could have appeared to influence the work reported in this paper.
